# Typology and correlates of parental stress among caregivers of children with DBDs in low-resourced communities in Uganda

**DOI:** 10.1371/journal.pgph.0002306

**Published:** 2023-08-23

**Authors:** Rachel Brathwaite, Natasja Magorokosho, Flavia Namuwonge, Nhial Tutlam, Torsten B. Neilands, Mary M. McKay, Fred M. Ssewamala

**Affiliations:** 1 International Center for Child Health and Development, Brown School, Washington University in St. Louis, St. Louis, Missouri, United States of America; 2 Division of Prevention Science, University of California, San Francisco, San Francisco, California, United States of America; 3 Brown School, Washington University in St. Louis, St. Louis, Missouri, United States of America; Amoud University College of Health Sciences, SOMALIA

## Abstract

Disruptive Behavior Disorders (DBDs) is one of the most common mental health problems among children in Uganda and SSA. Yet, to our knowledge no research has studied parenting stress (PS) among caregivers of children with DBDs, or investigated which risk factors originate from the child, parent, and contextual environment. Using a rigorous analytical approach, we aimed to: 1) identify different types and; 2) examine factors associated with PS and how correlates differ according to the type of stress experienced among caregivers of children with DBDs in low-resourced Ugandan communities. We used data from 633 caregivers of children with DBDs from SMART-Africa Uganda study. PS, was measured using the 36-item Parenting Stress Index-Short Form (PSI-SF). To identify focal correlates related to child/parent/contextual environment, we performed variable importance screening using the Stata command -gvselect- and specified mixed/melogit multilevel modeling with random effects. Secondly, focal correlates were included in the cross-fit partialing out lasso linear/logistic regression (double machine-learning) model. Caregivers mostly experienced stress from parental distress and caring for a child with difficult behavior. As scores increased by one unit on: caregiver mental health distress, PSI-SF increased by 0.23 (95% CI = 0.15, 0.32) (reflecting higher stress levels); Child difficulties, PSI-SF increased by 0.77 (95% CI = 0.52, 1.02). Contrastingly, for every one unit increase in family cohesion scores, PSI-SF decreased by 0.54 (95% CI = -0.84, -0.23). Caregivers with college/diploma/undergraduate/graduate education had less stress than those completing primary only or never attended school [Coefficient = -8.06 (95% CI = -12.56, -3.56)]. Family financial supporters had significantly higher Parental distress than caregivers who were not [Coefficient = 2.68 (95% CI = 1.20, 4.16)]. In low-resource settings like Uganda where mental health support is limited, community-based family-focused and economic empowerment interventions that improve community support systems and address financial barriers can reduce stress levels of caregivers of children with DBDs.

## Background

Parenting stress is defined as an individual’s perception of difficulty in achieving the expected demands of the parenting role [1]. Parents are overburdened when the demands of parenting outweigh the resources available to satisfy those expectations [2]. A higher degree of parental stress is associated with higher levels of psychological discomfort in parents [3] and worse developmental outcomes in children [4, 5]. Furthermore, highly stressed parents or caregivers exhibit more maladaptive parenting behaviors, such as less sensitivity and responsiveness to their children’s needs [4], higher criticality, less warmth, and more rejection, than their less stressed counterparts [2, 5–7]. Parental stress is also linked to harsher discipline, greater control over child conduct, and an increased likelihood of child maltreatment [8].

Indeed parenting is challenging, but the stressors can be intensified when residing in poverty-impacted environments [9], and elevated when raising children with behavioral problems [10]. In Uganda, a low-income country in sub-Saharan Africa (SSA), more than half of children and adolescents are being raised in communities affected by multi-dimensional poverty and low standards of living [11]. Poverty and financial uncertainty are major factors that contribute to parenting stress. Parents living in poverty encounter numerous difficulties and making decisions about how to juggle limited resources can increase their stress levels [12]. Along these same lines, disruptive behavioral disorders (DBDs) can disrupt parent-child relationships and contribute to parental stress [13]. Disruptive Behavior Disorders (DBDs) and are defined by symptoms of inattention, impulsivity, and hyperactivity [14]. DBDs are common among children and adolescents in SSA with high rates of comorbid mood, anxiety, substance use, and learning disorders [15]. DBDs negatively affect the mental health functioning of children and adolescents, and without early intervention, DBDs are likely to progress into adulthood, resulting in dire consequences.

Children residing in poverty are four times more likely to experience DBDs than youth in resource-rich communities [16]. Given that in Uganda, many communities are heavily impacted by social problems including chronic poverty (38%), domestic violence (30%), and physical violence against children (80%), as well as diseases like malaria (70 to 80%) and HIV/AIDS (12%) [17–20], these can greatly increase the stressors of daily life and increase the risk of DBDs among children. Other risk factors such as social isolation, high stress, and lack of social support are prevalent in Uganda, which can influence parenting and the development of behavioral problems in children [21]. Furthermore, there is a low availability of mental health professionals in Uganda to meet the growing child and adolescent mental health needs, hence the burden of DBDs is increasing in Uganda [22].

DBDs are one of the most common mental health problems among children in SSA, including Uganda [23]. Caring for children with DBDs can be a source of parental stress since children with DBDs often display aggression, defiance, rule-breaking and disruptive behaviors which are challenging to manage and can create family conflict [24]. This can place an emotional strain on parents as they experience feelings of guilt, frustration, and worry about being able to manage their child’s behavior, and their success in school, their ability to maintain healthy relationships and their future [24]. Parents may also experience stress due to feeling isolated or stigmatized, be stressed about the resulting impact of their child’s behavior on family dynamics, experience financial stress from seeking treatment, and become generally stressed due to the substantial additional cost to their time and energy [24]. Excessive stress caused by caring for children with DBDs in-turn affects parents’ ability to manage their own reactions and moods, which perpetuates poor mental health amongst themselves and problematic behavior and poor mental health in their children.

Additionally, parental stress is associated with several other factors that can be attributed to the child, parent, or contextual environment in which they live. Research has identified three areas that influence parenting: (a) child attributes such as personality and health, (b) parent characteristics such as their own mental health status [25], and (c) factors related to the local contextual environment in which they reside, such as family income, education level of parents/caregivers, marital status, family structure, race/ethnicity, and social support [26]. However, the majority of research on parenting stress has been conducted in high-income countries, and rarely focused on populations in SSA countries. Among the few research conducted in SSA, sample populations focused on specific high-risk populations including caregivers of AIDS-orphaned children [27], caregiving grandparents of AIDS-orphaned children [28, 29], caregivers of children living with HIV [30]. Furthermore, these stressors originating from the child, parent, and contextual environmental level have not been explored among caregivers of children with DBDs in Uganda where the burden is increasing. A recent systematic review published in 2022 that examined parent, child and situational factors associated with parenting stress identified only two studies conducted in SSA (Ghana and Côte d’Ivoire) [31, 32]. In both of these the target population included only urban mothers of young children. Given the potential negative consequences of parental stress, such as poorer parenting behavior, long-term problems affecting parent-child interaction [33], and child behavioral maladjustment [34], there is a need for more research to better understand which attributes of the child, parent, and contextual environment contribute to parenting stress among parents caring for children with DBDs in low-resourced communities like Uganda. In addition to understanding the correlates of parenting stress, it is also important to disaggregate and identify the different types of stress parents/caregiving families of children with DBDs in Uganda experience and how the correlates differ accordingly.

Thus, using a rigorous analytic approach the aims of this study are: 1) to identify the different types of parental stress experienced among caregivers of children with DBDs residing in low-resourced communities in southwestern Uganda and; 2) to examine the factors associated with different types of parental stress and how the correlates differ according to the type of stress experienced. Our findings may improve understanding of how we can support parents and caregiving families of children with DBDs to reduce their stress levels and better care for their children.

## Data and methods

### Study design and setting

This paper is based on a cross-sectional study design using baseline data from the SMART-Africa Uganda study, which was conducted in 26 public primary schools in five districts (Kalungu, Kyotera, Lwengo, Masaka, and Rakai) in the greater Masaka region of Southwestern Uganda [35] (S1 Data). Initially 30 schools were enrolled to participate, however the resulting social distance restrictions which included school closures, bans on gatherings, restrictions on intra-district travel, and to ensure safety of the participants and the research team, implemented during the COVID-19 pandemic prevented recruitment activities from commencing in 4 schools. Hence, data collection stopped at 26 schools in total (10 control schools, 8 schools with Amaka-parents, and 8 schools with Amaka-community). The schools are located in poverty-impacted communities exposed to numerous stressors, including high burden of HIV/AIDS, orphanhood, domestic violence, violence toward children, and depression [35]. These communities are considered at high risk for behavioral problems among youths. Located approximately 100 miles from the capital Kampala, the region is considered a mixture of rural (44%), semi-urban (45%) and urban (11%) [36].

The main objective of the SMART-Africa study was to test the effectiveness of a culturally-adapted multiple family group (MFG) intervention called Amaka Amasanyufu on reducing DBDs among children. A study protocol containing details of the SMART-Africa Uganda study has been published [35]. The research team undertook a thorough cultural adaptation process to ensure the Amaka Amasanyufu intervention was culturally appropriate for the Ugandan setting. The details of this process is described elsewhere [37]. The inclusion criteria for children within each school included: 1) currently in grades two to seven and ages 8–13 years; 2) caregiver completion of a screening questionnaire which assessed DBDs in their child. The three assessments included: i) Disruptive Behavior Disorder Rating Scale [38]; ii) Iowa Conners Scale [39], and iii) Impairment Rating Scale [40]; 3) caregiver provided written consent and adolescent provided assent. A child was considered positive for DBD if he or she met the criteria for at least one of the three conditions above [41]. Screening criteria are available in S1 Appendix. Only students who screened positive for DBDs (n = 636) were included in our analysis.

### Description of outcome

Our main outcome was parenting stress, which was measured using the 36-item self-reported Parenting Stress Index-Short Form (PSI-SF) [42]. The PSI-SF has been previously used to assess stress among adult populations in SSA [28], including Uganda [27], and has high internal consistency, Cronbach’s alpha = 0.88. The PSI-SF captures overall stress as well as three different domains/typologies of parental stress: 1) Parental Distress (PD); 2) Parent-Child Dysfunctional Interaction (P-CDI) and; 3) Difficult Child (DC) [42, 43]. Caregivers were required to rate each item on a 5-point scale, with higher raw scores indicating higher levels of stress. The PD domain assessed caregivers’ stress levels that originated from their feelings of being competent, restricted, supported, and/or depressed in their parenting role. For example, caregivers were asked to rate their agreement with statements such as “I feel trapped by my responsibilities as a parent” and “Since having this child I have been unable to do new and interesting things”. The P-CDI domain assessed the extent that caregivers felt satisfied with their child and their parent-child interactions. Example statements included “My child smiles at me much less than expected” and “Sometimes my child does things to bother me just to be mean”. On the other hand, the DC domain measured caregivers’ perception of how easy or difficult it is to take care of their child. For example, “My child turned out to be a bigger problem than expected” and “My child seems to cry or fuss more often than most children”. In this study we examined correlates of overall stress as well as each of the three stress subtypes.

### Correlates

#### Child factors

Biological sex, age, and child difficulties were included as child factors. Child’s age and child difficulties were treated as continuous variables. Child difficulties were evaluated using a 7-item scale. Caregivers were required to state whether a child’s difficulties affected/interfered with the child themselves, child’s family life, child’s friendships, child’s classroom learning, child’s leisure activities, the family as a whole, and the child’s home life. Response options ranged from Never = 1 to Always = 5, with higher scores indicative of experiencing more child difficulties. This scale had high internal consistency, Cronbach’s alpha = 0.89.

#### Parent factors

For parent factors, we included caregiver’s biological sex, frequency of engagement in religious activities, and mental health functioning. Caregivers were asked to rate the frequency they attended a church or mosque. We grouped responses into three categories: almost never or only on holidays; less than once a week but more than just on holidays (e.g., Christmas, Easter); and almost every week. Clinically relevant psychological symptoms in caregivers were assessed using an adapted version of the 34-item Brief Symptom Inventory (BSI) [44]. This 34-item version of the BSI has been previously used to assess caregivers’ mental health in Uganda [45, 46]. The scale assesses nine dimensions of mental health functioning among caregivers, including somatization, obsession-compulsion, interpersonal sensitivity, depression, anxiety, hostility, phobic anxiety, paranoid ideation, and psychoticism. Caregivers were required to select the intensity with which they experienced each symptom ranging from 1 = Never true to 5 = Always true. All items were summed to produce a total score with higher values representing higher levels of mental health distress. In this sample, BSI has a high internal consistency; Cronbach’s alpha of 0.92.

#### Contextual factors

**Fig 1:**
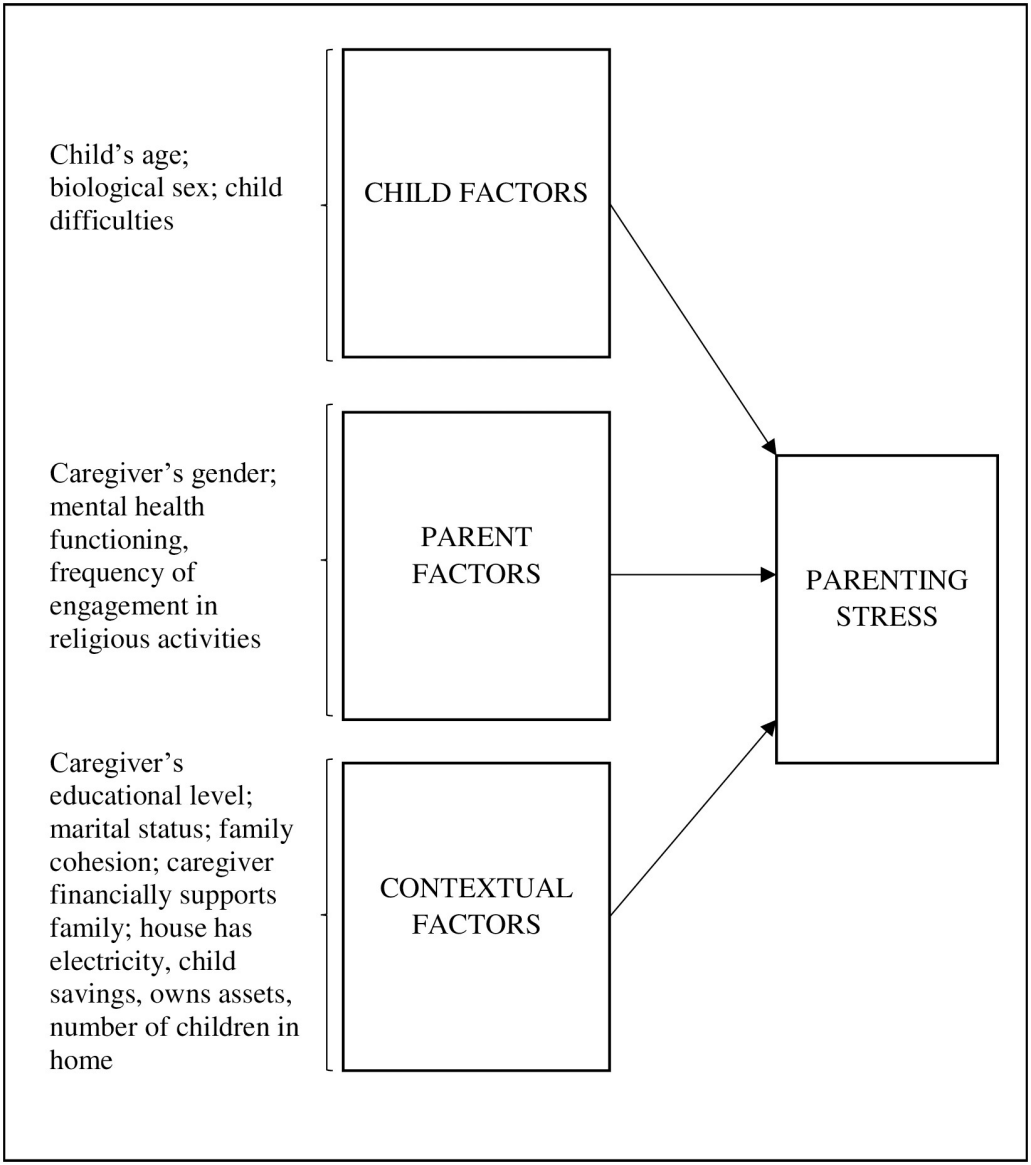
Conceptual framework (Adapted from Abidin, 1995).

Caregivers’ educational level, family cohesion, family structure, whether caregiver is responsible for financially supporting the family (yes/no), asset ownership, home has electricity, and number of children in the household were considered as contextual factors. The highest level of education completed by the caregiver was categorized into three groups: never attended school or primary level; all or part of secondary level; or college/diploma/undergraduate degree/graduate level. Family cohesion was assessed by an 8-item scale adapted from the Family Environment Scale [47] and Family Assessment measure [48], which assessed the degree of commitment, help, and support family members provided for one another. Caregivers were asked to rate how often family members: ask each other for help before asking non-family members; like to spend free time with each other; feel close to each other; are available to talk to other family members; listen to what other family members have to say, even in disagreements; or do things together as a family; take time to listen to the child when they want to talk; if the child has a problem, do they bring it to your attention so that you can help. Response options ranged from Never = 1 to Always = 5, with higher scores representing greater family cohesion. This scale had high internal consistency, Cronbach’s alpha = 0.74. As a proxy for financial resources, we assessed ownership of different types of assets including home/rental property/land ownership (yes/no) and whether caregivers had savings (had no savings at all/had no savings for the child/ had savings for the child). Our conceptual model, adapted from Abidin, 1995 [49] (Fig 1) depicts the relationships between the different factors and parental stress among caregivers in low-resource communities in Uganda.

### Ethical considerations

The SMART Africa-Uganda study was approved by the Washington University in St. Louis’s Institutional Review Board (#2016011088), the Uganda Virus Research Institute (GC/127/16/05/555), and the Uganda National Council of Science and Technology (SS4090). Study procedures were approved by the Data Safety and Monitoring Board at the National Institute of Mental Health. Written informed consent was obtained from all caregivers and assent was obtained separately from adolescents to avoid coercion prior to study enrolment.

### Inclusivity in global research

For additional information about ethical, cultural, and scientific considerations related to inclusivity in global research can be found in S1 Checklist.

### Data analysis

Categorical variables were summarized using counts and percentages while continuous variables were summarized using means and standard deviations (SD). To avoid the well-documented problems of stepwise variable selection [50], we performed an initial variable importance screening using the community-contributed Stata command -gvselect- to identify focal correlates [51]. Within -gvselect-, we specified the mixed and melogit multilevel modeling estimation command with random effects for continuous and binary outcomes, respectively. The log likelihood, Akaike’s information criterion (AIC), and the Bayesian information criterion (BIC) are reported for the best regressions at each of *k* correlates, along with the variable list of covariates from best *k* correlate model, where *k* is the number of correlates in the model. In the interest of finding the most parsimonious model, the best subset of focal correlates was chosen based on having the lowest Bayesian Information Criteria (BIC). In a second step, correlates were included in a lasso inference analysis using the cross-fit partialing out lasso linear and logistic regression (double machine-learning) method [52, 53] in Stata 17 [54]. The correlates identified in the screening step were included as focal correlates in the lasso analysis and the other non-selected covariates were controlled for, but their effects were not estimated. In all cross-fit partialing out lasso inference models, standard errors were adjusted for clustering by schools. To improve our understanding of sources of different types of stress caregivers experienced, we analyzed the three domains (PD, P-CDI, and DC) separately as well as the total stress score as separate continuous outcomes and examined whether the correlates differed for each domain of stress.

### Sensitivity analysis

Since BIC tends to favor more parsimonious models, to avoid missing potentially important correlates, as a sensitivity analysis we also evaluated whether the additional subset of correlates determined from the model with the lowest Akaike Information Criteria (AIC) were significant when added to the lasso inference model. Additionally, we used percentile scores to understand caregivers’ relative standing amongst all the caregivers in the sample. We calculated the proportion of caregivers with percentile scores above the threshold considered typical for each domain. Caregivers with percentile scores greater than 80 on all domains and on the total parental stress score were considered to be experiencing high stress levels [42]. We examined which type of stress was most common (i.e., which domain had the highest proportion of caregivers reporting high stress and which domain had the highest mean scores) and how the correlates of high stress differed according to the domain.

## Results

### Socio-demographic characteristics of participants

A total of 633 participants were included in this analysis. Three participants did not complete the full assessment at baseline, thus they were excluded from the analysis. The average age of children at baseline was 11.4 years, and ~52% were females. Most caregivers were also female (~83.4%), ~59% were married, ~10% were single, and ~31% were separated/divorced/widowed/other (Table 1). Primary caregivers of children with DBDs were included in the study. Among the primary caregivers, 69% (n = 439) were biological parents, 21.2% (n = 135) were grandparents, and 9.8% (n = 62) were other relatives. The majority of caregivers only completed primary school or never attended school (~69%). Over 70% attended church or mosque almost every week, the majority owned property (house, rental property or land) (~85%), lived in homes with electricity (~89%), and were financial supporters of the family (79%). On average, caregivers attributed the highest levels of stress to Parental Distress (mean scores = 37.8, SD = 8.2), followed by Difficult Child (mean = 34.9, SD = 6.1) then Parent-Child Dysfunctional Interaction (mean = 33.3, SD = 7.4). However, on examining the percentile scores (caregivers relative standing within all of the caregivers who were assessed), 21.3% experienced high stress related to the Difficult Child domain; 20.4% were experiencing high stress related to the Parental Distress domain, and 19.9% experienced high stress related to Parent-Child Dysfunctional Interaction domain. Among all caregivers: 19.1% were in the high stress category (above the 80th percentile) on the total parental stress score.

**Table 1:** Socio-demographic characteristics of study population.

Characteristics	Mean (SD)/ n (%)
Age (child)	11.4 (1.4)
Gender (child)	
Male	308 (48.4)
Female	328 (51.6)
**Child difficulties**	17.7 (6.3)
**Caregiver biological sex**	
Male (ref)	105 (16.6)
Female	528 (83.4)
**Primary caregiver**	
Biological parents	439 (69.0)
Grandparents	135 (21.2)
Other relatives	62 (9.8)
**Caregiver mental health**	78.7 (20.8)
**Caregiver highest level of education**	
Never attended school or Primary level (ref)	436 (68.9)
All or part secondary level	158 (25.0)
College/diploma/undergraduate/graduate	39 (6.2)
**Caregiver frequency of engagement in religious activities**	
Almost never or only holidays (ref)	37 (5.9)
Less than once a week but more than just on holidays	128 (20.2)
Almost every week	468 (73.9)
**Savings**	
No savings for child (ref)	167 (26.4)
Yes savings for child	68 (10.7)
Do not have any savings at all	398 (62.9)
**Family cohesion**	31.8 (4.3)
**Number of children in home**	4.6 (2.0)
**Caregiver marital status**	
Single	63 (9.9)
Married	372 (58.8)
Divorced, separated, widowed	198 (31.3)
**Caregiver financially supports family**	
No (ref)	135 (21.3)
Yes	498 (78.7)
**Owns house, rental property or land**	
No (ref)	92 (14.5)
Yes	541 (85.5)
**House has electricity**	
No (ref)	69 (10.9)
Yes	564 (89.1)

SD = Standard Deviation

### Correlates associated with overall parenting stress as measured by the Parenting Stress Index-Short form (PSI-SF)

With respect to overall parenting stress, four correlates were retained as significant in the final model selected using lowest BIC: mental health of caregivers, child difficulties, family cohesion, and caregiver education level (Table 2). For every one unit increase in BSI score (indicative of higher levels of mental health distress among caregivers), parenting stress scores increased by 0.23 (95% CI: 0.15, 0.32) (reflecting higher stress levels) (Table 3). Similarly for every one unit increase in child difficulties scores, parenting stress scores increased by 0.77 (95% CI: 0.52, 1.02). Contrastingly, for every one unit increase in family cohesion scores, parenting stress levels decreased by 0.54 (95% CI: -0.84, -0.23). Caregivers who completed college/diploma/undergraduate or graduate level education had lower stress levels than those completing primary level only or never attended school [Coefficient = -8.06 (95% CI: -12.56, -3.56)]. None of the additional six correlates selected via AIC when added to the model were significant.

**Table 2:** List of correlates included in the optimal models selected using the lowest value of Bayesian Information Criteria (BIC) and Akaike Information Criteria (AIC), after fitting–gvselect- command for all outcomes (continuous outcomes).

Correlates	Parental Stress Index-Short Form (Total scale)		PD domain		P-CDI domain		DC domain	
	BIC	AIC	BIC	AIC	BIC	AIC	BIC	AIC
Age of child	-	-	-	-	-	-	-	-
Child’s biological sex	-	x	-	-	-	-	-	-
Child difficulties	x*	x*	x*	x*	x*	x*	-	x*
Caregiver biological sex	-	x	-	x	-	-	-	-
Caregiver mental health	x*	x*	x*	x*	-	-	x*	x*
Caregiver highest level of education	x*	x*	x*	x*	x*	x*	-	x*
Caregiver frequency of engagement in religious activities	-	x	-	-	-	-	-	-
Savings	-	-	-	-	-	-	-	-
Family cohesion	x*	x*	-	-	x*	x*	x*	x*
Number of children in home	-	-	-	-	-	-	-	-
Caregiver financially supports family	-	x	x*	x*	-	-	-	-
Owns house, rental property or land	-	x	-	x	-	x	-	x
House has electricity	-	x	-	x	-	-	-	x*
**Total covariates selected**	4	10	4	7	3	4	2	6

x = correlate was included in model after fitting–gvselect- command; x* = correlate was significant; - = correlate not included (non-focal correlate); AIC = Akaike information criterion; BIC = Bayesian Information Criterion

**Table 3:** Regression coefficients and 95% confidence intervals for focal covariates estimated using cross-fit partialing out lasso inference estimator for total parenting stress scale, and PD domain.

Correlates	Parental Stress Index-Short Form (Total scale)		Parental Stress Index-Short Form (Total scale)		Parental Distress domain (continuous)		Parental Distress domain (continuous)	
	BIC		AIC		BIC		AIC	
	Coef (95% CI)	p-value	Coef (95% CI)	p-value	Coeff (95% CI)	p-value	Coeff (95% CI)	p-value
**Child’s biological sex**								
Male (ref)			0					
Female			-0.16 (-2.85, 2.53)	0.906				
**Child difficulties**	**0.77 (0.52, 1.02)**	**<0.001**	**0.78 (0.53, 1.02)**	**<0.001**	**0.26 (0.14, 0.38)**	**<0.001**	**0.25 (0.13, 0.37)**	**<0.001**
**Caregiver biological sex**								
Male (ref)			0					
Female			2.77 (-0.46, 6.00)	0.093				
**Caregiver mental health**	**0.23 (0.15, 0.32)**	**<0.001**	**0.23 (0.15, 0.32)**	**<0.001**	**0.10 (0.14, 0.38)**	**<0.001**	**0.10 (0.06, 0.13)**	**<0.001**
**Caregiver highest level of education**								
Never or Primary level (ref)	0		0		0		0	
All or part secondary level	-1.26 (-3.72, 1.19)	0.311	-1.27 (-3.73, 1.20)	0.314	-0.25 (-1.48, 0.97)	0.685	-0.14 (-1.35, 1.06)	0.820
College/diploma/undergraduate/graduate	**-8.06 (-12.56, -3.56)**	**<0.001**	**-7.85 (-12,51, -3.19)**	**0.001**	**-5.05 (-7.35, -2.76)**	**<0.001**	**-4.63 (-6.90, -2.36)**	**<0.001**
**Caregiver frequency of engagement in religious activities**								
Almost never or only holidays (ref)			0					
Less than once a week but more than just on holidays			-1.27 (-6.66, 4.11)	0.643				
Almost every week			-3.00 (-8.03, 2.04)	0.243				
**Family cohesion**	**-0.54 (-0.84, -0.23)**	**0.001**	**-0.54 (-0.84, -0.23)**	**0.001**				
**Caregiver financially supports family**								
No (ref)			0		0		0	
Yes			2.77 (-0.46, 6.00)	0.093	**2.68 (1.20, 4.16)**	**<0.001**	**2.58 (1.12, 4.05)**	**0.001**
**Owns house, rental property or land**								
No (ref)			0				0	
Yes			0.01 (-3.39, 3.41)	0.997			-0.98 (-2.41, 0.45)	0.180
**House has electricity**								
No (ref)			0				0	
Yes			0.45 (-2.10, 3.00)	0.730			-0.94 (-2.41, 0.45)	0.180

Standard errors are adjusted for accounting for 26 clusters at the school level; AIC = Akaike information criterion; BIC = Bayesian Information Criterion; CI = confidence interval; Each lasso model adjusted for the corresponding non-focal covariates identified in Table 2 but their effects were not estimated. Bolded values are significant at the 0.05 level.

### Correlates associated with the parental distress domain

Four correlates were retained as significantly associated with caregivers feeling incompetent, restricted, unsupported, and/or depressed in their parenting role according to BIC (Table 2). Consistent with the findings thus far, for every one unit increase in child difficulties scores, parental distress scores increased by 0.26 (95% CI: 0.14, 0.38) and for every one unit increase in BSI scores (indicative of poorer caregiver mental health), parental distress scores increased by 0.10 (95% CI: 0.14, 0.38) (Table 3). Caregivers who were financial supporters of the family had significantly higher stress levels than caregivers who were not [Coefficient = 2.68 (95% CI: 1.20, 4.16)]. However, caregivers with higher educational level (college, diploma, undergraduate, or graduate) had lower stress levels Coefficient = -7.85 (95% CI: -12.51, -3.19) than caregivers with primary level education only or never attended school. The three additional correlates selected via AIC were not significant when added to the model (Tables 2 and 3).

### Correlates associated with the parent-child dysfunctional interaction domain

Correlates of parent-child dysfunctional interaction, included child difficulties, caregiver education level, and family cohesion (Table 2). As child difficulties scores increased by one unit, P-CDI scores increased by 0.13 (95% CI: 0.03, 0.25), indicative of more dissatisfaction with their child-parent interactions (Table 4). On the other hand, caregivers with higher education levels displayed significantly lower levels of stress due to their parent child interactions than caregivers who never attended school or only attended primary school [Coefficient = -2.27 (-4.33, -0.22)]. Family cohesion appeared to be protective. As family cohesion scores increased by one unit, dissatisfaction with parent-child interactions significantly decreased by 0.23 [Coefficient = -0.23 (95% CI: -0.37, -0.09)]. The additional correlate (ownership of house/rental property/land) identified from the AIC was not significant when added to the model (Table 4).

**Table 4:** Regression coefficients and 95% confidence intervals for focal correlates estimated using cross-fit partialing out lasso inference estimator for P-CDI and DC domains.

Correlates	Parent-Child Dysfunctional Interaction domain (continuous outcome)		Parent-Child Dysfunctional Interaction domain (continuous outcome)		Difficult Child domain (continuous outcome)		Difficult Child domain (continuous outcome)	
	BIC		AIC		BIC		AIC	
	Coeff (95% CI)	p-value	Coeff (95% CI)	p-value	Coeff (95% CI)	p-value	Coeff (95% CI)	p-value
**Child difficulties**	**0.13 (0.03, 0.25)**	**0.016**	**0.14 (0.03, 0.25)**	**0.011**			**0.16 (0.08, 0.26)**	**<0.001**
**Caregiver mental health**					**0.05 (0.02, 0.08)**	**0.001**	**0.05 (0.02, 0.08)**	**0.001**
**Caregiver highest level of education**								
Never or Primary level (ref)	0		0				0	
All or part secondary level	-1.03 (-2.26, 0.19)	0.099	-1.04 (-2.27, 0.19)	0.099			-0.30 (-1.28, 0.68)	0.548
College/diploma/undergraduate/graduate	**-2.27 (-4.33, -0.22)**	**0.030**	**-2.21 (-4.26, -0.16**	**0.035**			**-1.78 (-3.28, -0.27)**	**0.021**
**Family cohesion**	**-0.23 (-0.37, -0.09)**	**0.001**	**-0.23 (-0.37, -0.09)**	**0.001**	**-0.20 (-0.31, -0.09)**	**<0.001**		
**Caregiver financially supports family**								
No (ref)							0	
Yes							0.70 (-0.48, 1.88)	0.245
**Owns house, rental property or land**								
No (ref)			0					
Yes			1.51 (-2.27, 0.19)	0.099				
**House has electricity**								
No (ref)							0	
Yes							**0.87 (0.02, 1.72)**	**0.046**

Standard errors are adjusted for accounting for 26 clusters at the school level. AIC = Akaike information criterion; BIC = Bayesian Information Criterion; CI = confidence interval; Each lasso model adjusted for the corresponding non-focal correlates identified in Table 2, but their effects were not estimated. Bolded values are significant at the 0.05 level.

### Correlates associated with the difficult child domain

Two correlates were significantly associated with stress due to difficult child. A higher level of family cohesion was associated with lower stress levels on the DC domain [Coefficient = -0.20 (95% CI: -0.31, -0.09)] (Table 4). On the other hand, as BSI scores increased, stress attributed to difficult child domain increased [Coefficient = 0.05 (95% CI: 0.02, 0.08)]. In this instance, three of the four additional correlates identified via AIC were significant when added to the lasso inference model. When added to the model, child difficulties [(Coefficient = 0.16 (95% CI: 0.08, 0.26)] and house had electricity [Coefficient = 0.87 (95% CI: 0.02, 1.72)] was associated with significant increase in stress levels, while more educated caregivers had significantly lower stress levels than less educated caregivers [Coefficient = -1.78 (95% CI: -3.28, -0.27)].

### Correlates associated with high stress (scoring above 80^th^ percentile) in each domain

With respect to having a stress score above the 80^th^ percentile, indicative of relatively higher levels of stress, no new correlates were identified (S1 Table). The odds of having high stress (above the 80^th^ percentile) related to parental distress increased by 10% for every one unit increase in child difficulties (95% CI: 1.05, 1.15) and 3% for every one unit increase caregiver mental health scores (95% CI: 1.01, 1.04) (S2 Table). Caregivers who were financially responsible for the household had 2.29 times (95% CI: 1.29, 4.06) the odds of high stress compared to those who were not. For high stress related to parent-child dysfunctional interaction, only caregivers’ mental health was significantly associated (OR = 1.02; 95% CI 1.002, 1.03). For high stress attributed to children with difficult behavior, child difficulties score (OR = 1.08; 95% CI 1.03, 1.13), worse caregiver mental health scores (OR = 1.02; 95% CI 1.01, 1.03) and house had electricity increased odds of high stress (OR = 1.87; 95% CI 1.18, 1.21), while caregivers with post-secondary educational level were at 81% lower odds of being in the high stress category (OR = 0.19; 95% CI 0.04, 0.98) (S3 Table). For DC domain, the AIC identified family cohesion as an additional potentially important covariate: the odds of high stress was significantly reduced by 5% as family cohesion scores increased (OR = 0.95; 95% CI 0.91, 0.98).

## Discussion

This paper examined the correlates of parenting stress among caregivers of children with DBDs in poverty-impacted communities in Uganda and how the correlates differed according to the domain of stress. Our findings showed that caregivers were mostly experiencing stress arising from parental distress and caring for a child with difficult behavior. This may imply that in our sample feelings of being competent, restricted, supported, and/or depressed in their parenting role may be more burdensome than the feelings captured in the other domains. Parenting stress among caregivers of children with DBDs was linked to multiple sources related to the child (child difficulties), parent (mental health), and local contextual environment (caregivers’ educational level, family cohesion, financial supporter of the family, and electricity in the home). We observed the correlates differed according to the type of stress experienced. Unsurprisingly, child difficulties score was a common risk factor associated with an increase in overall stress as well stress related to the caregiver’s personal adjustment to parenthood (PD domain), stress originating from having a hard time getting their child to cooperate and/or managing their child’s behavior (DC domain), and stress related to their feelings of disappointment, rejection, or alienation by/from the child, or a lack of proper bonding with their child (P-CDI domain). For the DC and PD domains as well as for overall stress, caregivers’ mental health was identified as a significant risk factor, with poorer mental health associated with higher levels of parenting stress. Among the remaining child factors, only biological sex was identified as a potential correlate according to the AIC, but was not significant. Among the potential contextual factors, we found that caregivers with post-secondary level education, and families with stronger family cohesion, were protective against stress whereas caregivers who supported the family financially and lived in homes with electricity showed higher levels of parental stress. Overall, our findings highlight the importance of improving mental health, strengthening family relationships and social support, and creating opportunities for continuing education and growing family financial resources as relevant for reducing parenting stress among caregivers of children with DBDs in low-resource settings. Interventions designed to improve these modifiable characteristics should be promoted in order to reduce stress levels and reduce negative outcomes in both caregivers and children with DBDs in Uganda.

Improving mental health of caregivers is important, since caregivers’ psychological wellbeing is a significant determinant of parenting quality and children’s wellbeing [55]. Caregivers who are experiencing mental health challenges will experience greater difficulty in caring for their child, fueling a cyclical relationship of poor mental health. The fact that stress levels decreased as family cohesion increased also speaks to the importance of social support networks that can help caregivers navigate the daily emotional and financial struggles of caring for children with DBDs. Similar research observed that caregivers with a weak support system had higher levels of depressive symptoms [56], strengthening the evidence base for interventions designed to strengthen family relationships. Our findings also highlighted the impact financial-related stressors had on caregivers’ stress levels. We observed those who were financially responsible for the family had higher stress levels. This aligns with previous research which observed greater psychological stress among persons unable to pay bills and afford basic necessities and identifies financial insecurity as the leading cause of psychological distress among adults seeking to provide for their families in Uganda [57]. Financial stress is associated with poor mental health, sleep problems [58], heart disease, and other chronic illnesses [59]. Given the high rates of unemployment prevalent in poverty-impacted environments, interventions that equip participants with asset building skills and knowledge on building a business are likely to have immense impact on overcoming some of these financial barriers. Along the same lines, we observed caregivers with higher education levels had significantly lower stress levels. Education plays an important role in getting access to emotional and financial resources to reduce their distress levels, since they are more likely to know how to circumvent and cope with adverse/challenging situations such as caring for children with DBDs [60]. Better educated caregivers are more likely to reach out for professional help when experiencing mental health problems, both personally and with their children. Thus, educational interventions that can equip caregivers with knowledge on how to effectively parent and communicate with children with behavioral disorders, and build positive parent-child relationships will prove vital in these settings, even among caregivers who are not highly educated.

Although our findings about parental stress among caregivers of children with DBDs are similar to factors observed in other populations, there were also unexpected findings. Caregivers residing in homes with electricity had higher stress levels and increased risk of high stress related to managing difficult child behavior. It might be that although these caregivers are able to afford electricity in their homes, dealing with challenging behavior and being unable to gain cooperation from their children may be too taxing and compound the stress levels. There may be a higher level of self-expectation for successful parenting when the basic needs have already been met. Along similar lines, it might be that less resourced parents may be so focused on basic survival for themselves and their families that they do not have time to focus on parenting stressors. Specific interventions on age-appropriate discipline and strategies to handle difficult behavior may be beneficial [42].

Our findings should be interpreted in line with the limitations of this analysis. This was a cross-sectional analysis and so we cannot determine causality. Additionally, all measures were self-reported and thus caregivers may try to minimize any problems to appear competent. However, we did not have any abnormally low levels of stress (<10 on raw total score), so it appears that caregivers were forthcoming in their responses. Finally, our findings cannot be generalized to other caregivers with children not displaying disruptive behavior disorders, or of older or younger ages, or residing in more urban settings, or of higher socioeconomic status. However, there were many strengths. We utilized robust analysis methods including -gvselect- to select the focal covariates, which overcomes the limitations of stepwise methods as it uses the leaps-and-bounds algorithm of Funival and Wilson [61], which is a more dependable algorithm, as it gives the best model for each correlate/covariate quantity by examining a manageable fraction of all possible models. We were able to estimate the coefficients and standard errors for our focal covariates using the cross-fit partialing out lasso inference methods with the cluster option, while adjusting for a set of control variables. This ensured the estimates represented values from the true model that generated the data being analyzed [53].

## Conclusion

Caregivers of children with DBDs in Uganda are exposed to numerous factors that increase their risk of stress related to parental distress, parent-child dysfunctional interaction and having a child who presents behavioral challenges (i.e., a “difficult” child). Caregiver mental health and child difficulties were risk factors of overall stress. However, we saw protective effects of family cohesion, and education on caregiver stress levels. Given the correlates identified in this analysis, in low-resource setting such as Uganda where mental health support is limited, community-based family-focused and economic empowerment interventions that seek to improve community support systems and address financial barriers can go a long way in reducing stress levels of caregivers of children with disruptive behavior disorders.

## Supporting information

S1 Checklist Inclusivity in global research. (DOCX)

S1 Data Study dataset. (SAV)

S1 Appendix Description of criteria and rationale of the measures used to assess DBDs among children. (DOCX)

S1 Table List of correlates included in the optimal model for high stress selected using the lowest value of Bayesian Information Criteria BIC and Akaike Information Criterion AIC, after fitting–gvselect- command for binary outcomes. (DOCX)

S2 Table Coefficients and 95% confidence intervals for focal correlates estimated using cross-fit partialing out lasso inference estimator for high stress on parental distress and parent-child dysfunctional interaction domains. (DOCX)

S3 Table Coefficients and 95% confidence intervals for focal correlates estimated using cross-fit partialing out lasso inference estimator for high stress on difficult child domain. (DOCX)

## Abbreviations

BSI: Brief Symptom Inventory
DBDs: Disruptive Behavior Disorders
DC: Difficult Child
MFG: Multiple Family Group
P-CDI: Parent-Child Dysfunctional Interaction
PD: Parental Distress
PSI-SF: Parenting Stress Index-Short Form
SMART-Africa: Strengthening Mental Health and Research Training in Africa
