# Supplementary material for: Typology and correlates of parental stress among caregivers of children with DBDs in low-resourced communities in Uganda
Source: PLOS Glob Public Health. 2023 Aug 23;3(8):e0002306. doi: 10.1371/journal.pgph.0002306 (PMC10446180; doi:10.1371/journal.pgph.0002306)
Supplement: S1 Checklist — (DOCX) [file pgph.0002306.s001.docx]

Inclusivity in global research

PLOS’ policy on inclusivity in global research aims to improve transparency in the reporting of research performed outside of researchers’ own country or community and ensures that PLOS publications reporting global research adhere to high standards for research ethics and authorship. Authors of relevant research articles may be asked to complete the questionnaire below, which outlines ethical, cultural, and scientific considerations specific to inclusivity in global research. This questionnaire may be requested when researchers have travelled to a different country to conduct research, if research uses samples collected in another country, research with Indigenous populations or their lands, or if research is on cultural artefacts. Researchers travelling to another country solely to use laboratory equipment will not normally be required to complete the questionnaire. However, the questionnaire can be requested at the journal’s discretion for any submission – if you have been requested to complete this questionnaire by the PLOS journal you submitted to, please do so.

Please complete the questionnaire below and include this as a Supporting Information file with your manuscript. Note that if your paper is accepted for publication, this checklist will be published with your article in the supporting information files. Please ensure that you reference the checklist in the main body of your manuscript. We suggest adding a subsection ‘Inclusivity in global research’ to your Methods section and adding the following sentence: “Additional information regarding the ethical, cultural, and scientific considerations specific to inclusivity in global research is included in the Supporting Information (SX Checklist)”

The questions have been designed to be applicable to a wide range of study types, and there are subsections for both human subjects research and non-human subjects research. If any of the questions are not relevant to your research please mark them as “N/A” as appropriate.

**Ethical considerations, permits and authorship**

*This section is applicable to all research types.*

Provide details as to who granted permissions and/or consent for the study to take place in the Methods section of your manuscript. This should include the names of **all** ethics boards, governmental organizations, community leaders or other bodies that provided approval for the study. If individuals provided approval refer to these people by their role or title but do not list their name(s).

Reported on page number: page 12

The SMART Africa-Uganda study was approved by the Washington University in St. Louis’s Institutional Review Board (#2016011088), the Uganda Virus Research Institute (GC/127/16/05/555), and the Uganda National Council of Science and Technology (SS4090). Study procedures were approved by the Data Safety and Monitoring Board at the National Institute of Mental Health.

If there were any deviations from the study protocol after approval was obtained please provide details of these changes in the Methods section of your manuscript.
Did this study involve local collaborators that are residents of the country where the research was conducted or members of the community studied? If you do not have any authors from said communities, please provide an explanation for this below.

The text on page 6 describes changes in the planned number of schools to be recruited due to the nation-wide school closures to prevent the spread of COVID-19.

*Initially 30 schools were enrolled to participate, however the resulting social distance restrictions which included school closures, bans on gatherings, restrictions on intra-district travel, and to ensure safety of the participants and the research team, implemented during the COVID-19 pandemic prevented recruitment activities from commencing in 4 schools. Hence, data collection stopped at 26 schools in total (10 control schools, 8 schools with Amaka-parents, and 8 schools with Amaka-community). p(6)*

Reported on page number: 7-8

*This study was a collaboration between Washington University in St. Louis and the International Center for Child Health and Development (ICHAD) Uganda. Flavia Namuwonge is a Ugandan and is a team member (research director and study coordinator) of ICHAD Uganda and Fred Ssewamala is a Ugandan and the Director of ICHAD Uganda. Flavia’s affiliation is listed as Washington University in St. Louis since she has recently enrolled as a PhD student at Washington University in St. Louis, however she coordinated the data collection during the time that the study was being implemented in Uganda.*

Everyone listed as an author should meet PLOS’ criteria for authorship and all individuals who meet these criteria should be included in the author byline, rather than the acknowledgements. Authorship criteria is based on the International Committee of Medical Journal Editors (ICMJE) Uniform Requirements for Manuscripts Submitted to Biomedical Journals - for further information please see here: <https://journals.plos.org/plosone/s/authorship>.

**Human subjects research (e.g. health research, medical research, cross-cultural psychology)**

Did you obtain written informed consent from a representative of the local community or region before the research took place? How did you establish who speaks for the community? Details of written informed consent obtained from study participants should be reported separately in the Methods section of your manuscript.

Written informed consent was obtained from all caregivers and assent was obtained separately from adolescents to avoid coercion prior to study enrolment.

This is reported on page 12 in the manuscript.

How did members of the local community provide input on the aims of the research investigation, its methodology, and its anticipated outcome(s)?

When engaging with the local community, how did you ensure that the informed consent documents and other materials could be understood by local stakeholders?

The main objective of the SMART-Africa study was to test the effectiveness of a culturally-adapted multiple family group (MFG) intervention called Amaka Amasanyufu on reducing DBDs among children. Prior to doing so the research team undertook a thorough cultural adaptation process to ensure the Amaka Amasanyufu intervention was culturally appropriate for the Ugandan setting. The details of this process is described in the paper published in the Family Process journal: *‘Sensoy Bahar O, Byansi W, Kivumbi A, et al. From "4Rs and 2Ss" to "Amaka Amasanyufu" (Happy Families): Adapting a U.S.-based Evidence-Based Intervention to the Uganda Context. Fam Process. 2020;59(4):1928-1945.’*

By engaging the local community in this way, we ensured the local community approved the methods and the intervention was culturally appropriate.

In addition, all questionnaires and intervention materials were translated into Luganda (language spoken in the study region) and back translated into English to ensure accuracy. This process was overseen by certified language experts at the Makerere University institute of languages in Uganda and administered in the local language Luganda, ensuring the materials were understood by local stakeholders.

Will the findings of the research be made available in an understandable format to stakeholders in the community where the study was conducted (e.g. via a presentation, summary report, copies of publications, etc.)? Please provide details of how this will be achieved.

Yes, all findings will be disseminated to the local community stakeholders using various formats. The research team will organize stakeholder meetings for teachers, caregivers, health care workers, religious leaders, political leaders and participants to share study progress, outcomes and challenges as well as receive feedback from the community members. ICHAD has established strong collaborations with researchers, NGOs, and government officials in Uganda and the U.S. who come with a wide range of research, policy, and practice expertise. ICHAD and SMART Africa Center have held annual conferences on child behavioral health and in Sub Saharan Africa (SSA) both on the African continent and in the United States. The meetings have been officiated by government officials and NIH representatives. Both centers provide a vibrant hub that shares evidence and collaborate on integrative and contextually specific programs and solutions for a range of social issues affecting children in the SSA region.

Annual reports and monthly newsletters highlighting the progress of multiple NIH-funded studies are published and shared with collaborators, center affiliates, and community partners. The reports are shared electronically with over 500 individuals on our listserv and made available on our website (https://ichad.wustl.edu/reports/). In addition, we provide print copies to our stakeholders in Uganda who may not have access to internet. In addition, monthly newsletters (Monthly Monitor) are shared with the listserv to provide timely updates on the progress of our studies and disseminate our findings. Our newsletters are also posted on our website at https://ichad.wustl.edu/ichad-updates/

**Non-human subjects research using specimens/ animals collected as part of the study, or those housed in archival collections. Examples include archaeology, paleontology, botany and zoology.**

Did the permission you obtained from a local authority to perform the study include an agreement on access to outputs and benefit sharing? This may include procedures to enable fair distribution of the benefits and resources arising from the research performed. Please include any details of Prior Informed Consent and Benefit Sharing Agreements obtained. These may be required by field-specific regulations, for example the Convention on Biological Diversity (CBD) and the associated Nagoya Protocol.

N/A

If the material used in your study was imported, please A) provide the year it was imported and B) indicate whether permits were obtained to import/export the materials used, C) provide details of any permits obtained. If this information is not available, please indicate this.

N/A

If you used archival specimens, please state how the material used in your study was acquired by the institute it is held in and provide details of any permits obtained for the original excavations/ sample collection. If this information is not available, please indicate this.

N/A

How was the potential cultural significance of the materials collected in your study to local communities considered in your research design? Were Indigenous peoples and/or local researchers and institutions involved with archaeological excavations / collection of specimens? If so, please provide a description of their involvement.

N/A

If your manuscript includes photographs of human remains please indicate whether authors obtained permission from descendants or affiliated cultural communities to do so.

N/A
