# Supplementary material for: Typology and correlates of parental stress among caregivers of children with DBDs in low-resourced communities in Uganda
Source: PLOS Glob Public Health. 2023 Aug 23;3(8):e0002306. doi: 10.1371/journal.pgph.0002306 (PMC10446180; doi:10.1371/journal.pgph.0002306)
Supplement: S1 Appendix — (DOCX) [file pgph.0002306.s003.docx]

**S1 Appendix**

**Description of criteria and rationale of the measures used to assess DBDs among children**

*Given the dearth of studies that focus on DBDs in sub-Saharan Africa, the study used multiple measures to estimate the full range of mental health needs among children. Hence, three measures were used to assess disruptive behavior challenges, with each measure capturing slightly different constructs. The three screening measures used were: 1) The Disruptive Behavior Disorder Rating Scale; 2) Iowa Conners Rating Scale and; 3) Impairment Scale. The Disruptive Behavior Disorder Rating Scale (Pelham, Fabiano, & Massetti, 2005) is a screening measure to detect oppositional defiant disorder and conduct disorder symptoms among children and adolescents. The scale contains 8 items that correspond to ODD symptoms and 15 items that are related to CD symptoms. Each item is evaluated on a four-point Likert scale (1 = Not at all, 2 = Just A Little, 3 = Pretty Much, 4 = Very Much). Examples of questions related to the ODD subscale include ‘Often actively defies or refuses to comply with adults’ requests or rules and items related to the CD subscale include Often stays out at night despite parental prohibitions. Items of the subscales were summated to ascertain the presence of DBDs (score ranges from 8-32 for ODD and 15-60 for CD) with sub-scales indicating acceptable levels of internal consistency (α=0.78 for ODD and α=0.71 for CD). To be considered positive for ODD, caregivers needed to endorse a total of four or more items as “pretty much” or “very much” when describing the behavior of their child. Likewise, to be considered positive for CD, caregivers had to endorse a total of three or more items in any category or any combination of categories as “pretty much” or “very much” when describing the behavior of their child.*

*The Iowa Conners scale is a short 10-item scale used to assess the severity of inattentive-impulsive overactive behavior (items 1-5) and oppositional defiant behavior (items 6-10) among children. We used only the ODD subscale of the Iowa Conners scale for this study. Parents who gave children scores of 6 or higher were categorized as screening positive for ODD (Pelham et al., 1989).*

*The Impairment Rating Scale comprised seven items. On a scale from 1 to 6 (where 1 = no problem and 6 an extreme problem), caregivers were required to select the point that they believed reflected the impact of the child's problems on this area and whether he or she needs treatment or special services for the problems. A score of 3 or higher in four or more items is considered a significant impairment. (p1 of Supplementary Materials)*

References

Pelham WE Jr., Milich R, Murphy DA, Murphy A. Normative Data on the IOWA Conners Teacher Rating Scale, Journal of Clinical Child Psychology, 1989;18:3, 259-262, doi: 10.1207/s15374424jccp1803_9

Pelham WE, Jr., Fabiano GA, Massetti GM. Evidence-based assessment of attention deficit hyperactivity disorder in children and adolescents. J Clin Child Adolesc Psychol. 2005;34(3):449-7
