# Supplementary material for: Typology and correlates of parental stress among caregivers of children with DBDs in low-resourced communities in Uganda
Source: PLOS Glob Public Health. 2023 Aug 23;3(8):e0002306. doi: 10.1371/journal.pgph.0002306 (PMC10446180; doi:10.1371/journal.pgph.0002306)
Supplement: S1 Table — (DOCX) [file pgph.0002306.s004.docx]

**S1 Table. List of correlates included in the optimal model for high stress selected using the lowest value of Bayesian Information Criteria (BIC) and Akaike Information Criterion (AIC), after fitting –gvselect- command for PD, P-CDI and DC domains (binary outcomes)**

| **Correlates** | **High stress on Parental Distress domain (above 80^th^ percentile)** | | **High stress on Parent-Child Dysfunctional Interaction domain**  **(above 80^th^ percentile)** | | **High stress on Difficult Child Domain**  **(above 80^th^ percentile)** | |
| --- | --- | --- | --- | --- | --- | --- |
|  | **BIC** | **AIC** | **BIC** | **AIC** | **BIC** | **AIC** |
| Age of child | - | - | - | - | - | - |
| Child’s biological sex | - | - | - | x | - | - |
| Child difficulties | x* | x* | - | - | x* | x* |
| Caregiver biological sex | - | - | - | - | - | - |
| Caregiver mental health | x* | x* | x* | x* | x* | x* |
| Caregiver highest level of education |  | x | - | - | x* | x* |
| Caregiver frequency of engagement in religious activities | - | - | - | - | - | - |
| Savings |  | x | - | - | - | - |
| Family cohesion | - | - | - | - | - | x* |
| Number of children in home | - | - | - | - | - | - |
| Caregiver financially supports family | x* | x* | - | - | - | - |
| Owns house, rental property or land | - | - | - | x | - | - |
| House has electricity | - | x | - | - | x* | x* |
| **Total covariates selected** | 3 | 6 | 1 | 3 | 4 | 5 |

x=correlate was included in the model; x*=correlate was significant; -=correlate not included (non-focal correlate); AIC= Akaike information criterion; BIC=Bayesian Information Criterion
