# Supplementary material for: Typology and correlates of parental stress among caregivers of children with DBDs in low-resourced communities in Uganda
Source: PLOS Glob Public Health. 2023 Aug 23;3(8):e0002306. doi: 10.1371/journal.pgph.0002306 (PMC10446180; doi:10.1371/journal.pgph.0002306)
Supplement: S2 Table — (DOCX) [file pgph.0002306.s005.docx]

**S2 Table. Coefficients and 95% confidence intervals for focal correlates estimated using cross-fit partialing out lasso inference estimator for High Stress on Parental Distress and Parent-Child Dysfunctional Interaction domains**

| **Correlates** | **High stress on Parental Distress domain (above 80^th^ percentile)** | | | | **High stress on Parent-Child Dysfunctional Interaction domain**  **(above 80^th^ percentile)** | | | |
| --- | --- | --- | --- | --- | --- | --- | --- | --- |
|  | **BIC** | | **AIC** | | **BIC** | | **AIC** | |
|  | **Odds ratio (95% CI)** | **p-value** | **Odds ratio (95% CI)** | **p-value** | **Odds ratio (95% CI)** | **p-value** | **Odds ratio (95% CI)** | **p-value** |
| **Child’s biological sex** |  |  |  |  |  |  | 1 |  |
| Male (ref) |  |  |  |  |  |  | 0.64 (0.40, 1.02) | 0.059 |
| Female |  |  |  |  |  |  |  |  |
| **Child difficulties** | **1.10 (1.05, 1.15)** | **<0.001** | **1.09 (1.04, 1.15)** | **<0.001** |  |  |  |  |
| **Caregiver mental health** | **1.03 (1.01, 1.04)** | **<0.001** | **1.03 (1.01, 1.04)** | **<0001** | **1.02 (1.002, 1.03)** | **0.026** | **1.02 (1.002, 1.03)** | **0.024** |
| **Caregiver highest level of education** |  |  |  |  |  |  |  |  |
| Never or Primary level (ref) |  |  | 1 |  |  |  |  |  |
| All or part secondary level |  |  | 0.95 (0.57, 1.58) | 0.837 |  |  |  |  |
| College/diploma/undergraduate/graduate |  |  | <0.01 (0, .) | 0.995 |  |  |  |  |
| **Savings** |  |  |  |  |  |  |  |  |
| No savings for child (ref) |  |  | 1 |  |  |  |  |  |
| Yes savings for child |  |  | 0.78 (0.28, 2.20) | 0.638 |  |  |  |  |
| Do not have any savings at all |  |  | 1.62 (0.90, 2.93) | 0.110 |  |  |  |  |
| **Caregiver financially supports family** |  |  |  |  |  |  |  |  |
| No (ref) | 1 |  | 1 |  |  |  |  |  |
| Yes | **2.29 (1.29, 4.06)** | **0.005** | **2.21 (1.17, 4.17)** | **0.014** |  |  |  |  |
| **Owns house, rental property or land** |  |  |  |  |  |  |  |  |
| No (ref) |  |  |  |  |  |  | 1 |  |
| Yes |  |  |  |  |  |  | 1.93 (0.78, 4.80) | 0.155 |
| **House has electricity** |  |  |  |  |  |  |  |  |
| No (ref) |  |  | 1 |  |  |  |  |  |
| Yes |  |  | 0.68 (0.57, 1.58) | 0.837 |  |  |  |  |

Standard errors are adjusted for accounting for 26 clusters at the school level; AIC= Akaike information criterion; Bayesian Information Criterion; CI= confidence interval; Each lasso model adjusted for the corresponding non-focal correlates identified in Table S1, but their effects were not estimated. Bolded values are significant at the 0.05 level.
