# Supplementary material for: Typology and correlates of parental stress among caregivers of children with DBDs in low-resourced communities in Uganda
Source: PLOS Glob Public Health. 2023 Aug 23;3(8):e0002306. doi: 10.1371/journal.pgph.0002306 (PMC10446180; doi:10.1371/journal.pgph.0002306)
Supplement: S3 Table — (DOCX) [file pgph.0002306.s006.docx]

**S3 Table. Coefficients and 95% confidence intervals for focal correlates estimated using cross-fit partialing out lasso inference estimator for High Stress on Difficult Child domain**

| **Correlates** | **High stress on Difficult Child domain (above 80^th^ percentile)** | | | |
| --- | --- | --- | --- | --- |
|  | **BIC** | | **AIC** | |
|  | **Odds ratio (95% CI)** | **p-value** | **Odds ratio (95% CI)** | **p-value** |
| **Child difficulties** | **1.08 (1.03, 1.13)** | **0.001** | **1.07 (1.02, 1.12)** | **0.001** |
| **Caregiver mental health** | **1.02 (1.01, 1.03)** | **<0.001** | **1.02 (1.01, 1.04)** | **<0.001** |
| **Caregiver highest level of education** |  |  |  |  |
| Never or Primary level (ref) | 1 |  | 1 |  |
| All or part secondary level | 0.76 (0.45, 1.26) | 0.285 | 0.80 (0.51, 1.26) | 0.340 |
| College/diploma/undergraduate/graduate | **0.19 (0.04, 0.98)** | **0.047** | **0.21 (0.06, 0.80)** | **0.022** |
| **Family cohesion** |  |  | **0.95 (0.91, 0.98)** | **0.007** |
| **House has electricity** |  |  |  |  |
| No (ref) | 1 |  | 1 |  |
| Yes | **1.87 (1.18, 1.21)** | **0.008** | **2.02 (1.29, 3.18)** | **0.002** |

Standard errors are adjusted for accounting for 26 clusters at the school level. AIC= Akaike information criterion; Bayesian Information Criterion; CI= confidence interval; Each lasso model adjusted for the corresponding non-focal correlates identified in Table S1, but their effects were not estimated. Bolded values are significant at the 0.05 level.
